# Mitigating Night Biomass Loss in Outdoor Pilot‐Scale Mixotrophic Algal Cultivation of *Monoraphidium minutum* Using Flue Gas Condensate and Cheese Whey

**DOI:** 10.1002/bit.70027

**Published:** 2025-07-18

**Authors:** Quyen Nham, Tristan Gordon, Hanna Farnelid, Catherine Legrand, Elin Lindehoff

**Affiliations:** ^1^ Department of Biology and Environmental Science Linnaeus University Centre for Ecology and Evolution in Microbial model Systems, Linnaeus University Kalmar Sweden; ^2^ School of Health and Welfare Jönköping University Jönköping Sweden

**Keywords:** cheese whey, flue gas condensate, *monoraphidium*; mixotrophic algal cultivation, night biomass loss, wastewater

## Abstract

In algal cultivation, nighttime biomass loss due to respiration and cell mortality can considerably reduce the amount of biomass produced during daylight. The adverse effect can be counteracted by mixotrophic cultivation, where an organic carbon (OC) source is used to supply the energy required for cell maintenance and division during darkness. The potential for mixotrophic cultivation to mitigate night biomass loss has yet to be tested under outdoor, large‐scale conditions that use raw industrial waste streams, particularly during low‐light seasons. We investigated night biomass loss in cultivation of the strain *Monoraphidium minutum* KAC90 in outdoor 1 m^3^ raceway ponds during the Nordic autumn. Flue gas condensate (nitrogen source) and cheese whey (phosphorus and OC source) were used for the mixotrophic treatment, while potassium monophosphate (phosphorus source) was used for the photoautotrophic control. Results indicate that under high OC availability, the mixotrophic treatment had a night biomass gain of 33% ± 16%, whereas it experienced a night biomass loss of 10% ± 9% under low OC. In contrast, the photoautotrophic control showed a night biomass loss of 5% ± 15%. In the mixotrophic treatment, algal biomass had a higher carbohydrate content, but lower levels of lipids and proteins than the photoautotrophic cultures. The cultivation of algae using cheese whey may increase biomass accumulation in darkness, enhancing the overall production of algal biomass rich in carbohydrates.

## 1 Introduction

Algal cultivation in wastewater is considered a promising method for bioremediation and recovery of nutrients to produce valuable biomass that can serve as feedstock for biofuel production in modern times (Jabłońska‐Trypuć et al. 2023). However, one of the challenges preventing this method's application to wastewater treatment is the low productivity of algal biomass (Kandasamy et al. 2023). Maintaining optimal levels of algal productivity is a key factor to improving the feasibility of commercializing algal cultivation technologies for applications such as wastewater treatment and biofuel production (Maroušek et al. 2023).

Photoautotrophic cultivation mode is both the earliest and most common technique used in algal farming (Zhan et al. 2017). In this mode, algal growth totally depends on light, and therefore biomass production ceases in darkness. As reported, most outdoor photoautotrophic cultures lose biomass during the night due to cellular dark respiration (Le Borgne and Pruvost 2013) and/or mortality (Le Borgne and Pruvost 2013). This phenomenon, defined as night biomass loss, is a significant factor influencing biomass productivity as it can reduce the total algal biomass by up to 53% overnight in photoautotrophic cultivation (Carneiro et al. 2020). The extent of night biomass loss varies with algal species, growth phase of the algae, temperature, daytime length and light intensity, as well as the duration of the darkness period. For instance, night biomass loss in exponential growth phase was more pronounced than in stationary phase (Edmundson and Huesemann 2015). Also, night biomass loss in algae is positively correlated with both nocturnal temperatures and the intensity of the light exposure preceding the night (Edmundson and Huesemann 2015; Tanaka et al. 2020).

Laboratory experiments with axenic cultures showed that night biomass loss could be effectively minimized by lowering the temperature, halting mixing during the night, or adding OC to stimulate heterotrophic growth (Ogbonna and Tanaka 1996). The mixotrophic cultivation mode is an attractive solution to mitigate night biomass loss while simultaneously increasing algal production efficiency, outperforming the limitations of both photoautotrophic and heterotrophic modes (Pang et al. 2019). Green algae are highly regarded candidates for industrial cultivation due to their robustness (Torres and De‐la‐Torre 2022). However, night biomass loss in green algal cultivation has predominantly been studied in photoautotrophic mode (Borovkov et al. 2019; Carneiro et al. 2020; de Vree et al. 2016; Edmundson and Huesemann 2015; Han et al. 2013; Hindersin et al. 2014; Holdmann et al. 2018; Nwoba et al. 2021; Xi et al. 2020). Few studies have investigated night biomass loss in mixotrophic mode, and these studies were primarily conducted under small‐scale laboratory conditions using axenic cultures and standardized media with pure OC sources, such as glucose, ethanol or acetate (Nair and Chakraborty 2020; Ogbonna and Tanaka 1996, 1998). A deeper understanding of night biomass loss in outdoor pilot‐ and large‐scale algal cultivation systems using organic materials from waste streams are essential for advancing the application of this method in industrial scale algal cultivation.

Flue gas condensate water (FGC) is a process water discharged from power plants. In many power plants, ammonia (NH_3_) is added to the boiler to catalyze the NO_x_ reduction reaction into N_2_ (Goldschmidt et al. 2015). Older fired plants often apply the reduction reaction without catalysts, requiring high temperature (900°C–950°C). The addition of NH_3_ enhances heat transfer and NO_x_ reduction, thereby lowering the required reaction temperature. However, unstable and non‐homogenized thermal levels in the boiler complicate accurate estimations of needed NH_3_ amount, often resulting in overdosing. Consequently, excess NH_3_ enters the flue gas and primarily ends up in the FGC as ammonium (NH_4_^+^), reaching concentrations up to 150 mg L^1^ (Goldschmidt et al. 2015), which requires treatment. Common methods for NH_4_^+^ removal, including absorption in quench, reverse‐osmosis filtration, gas membrane separation, and NH_3_ stripper, are expensive and unsustainable (Goldschmidt et al. 2015). Kalmar Energi power plant in Sweden produces 113–600 m^3^ day^−^^1^ FGC water that is treated by sand filtration, NH_3_ stripping, adsorption by activated carbon, and ion exchange (Gunnars and Magnusson 2020), in which nitrogen (N) is lost to the atmosphere. The potential for algal solutions to effectively recover NH_4_^+^ in FGC and convert it into biomass for subsequent applications remains largely unexplored.

Flue gas condensate water is deficient in phosphorus (P) and OC and requires an external supplementation to make up a nutrient‐balanced medium for mixotrophic algal cultivation. This study combined FGC with cheese whey, a wastewater from cheese processing that contains promising levels of P and OC, to formulate a medium for algae. Previous research demonstrated that whey permeate, a residual water of cheese whey after protein and fat removal, was a suitable source of P and OC for mixotrophic algae, both in laboratory settings and in outdoor pilot‐scale facilities when paired with landfill leachate as a N source (Nham et al. 2024; Nham et al. 2023). The green alga *Monoraphidium minutum* has been reported to grow well in diluted raw whey permeate (Nham et al. 2023). Meanwhile, cheese whey has been tested as a nutrient and OC source to support mixotrophic algal growth under controlled temperature and light conditions at laboratory scale (Casá et al. 2022; Borges et al. (2016); Kallarakkal et al. 2021; Pandey et al. 2020; Riaño et al. 2016). To the best of our knowledge, outdoor large‐scale algal cultivation using cheese whey remains unexplored, with limited studies investigating the potential of combining cheese whey with other waste streams to formulate a balanced medium for algae.

Bacterial contamination is unavoidable in outdoor, open cultivation systems. In mixotrophic cultivation, the use of organics, such as cheese whey, carries a high risk of bacterial development, which can outcompete algae for resources (Abiusi et al. 2020). However, bacteria in non‐axenic algal cultures have been reported to enhance algal growth, biomass production and nutrient removal from wastewaters (Fallahi et al. 2021). Thus, bacteria play a crucial role in outdoor algal cultivation for improving biomass yield and nutrient removal from wastewaters.

This study aims to investigate (1) night biomass loss in mixotrophic mode using wastewaters in outdoor pilot‐scale raceway open ponds, (2) the potential of using FGC‐cheese whey mixture for nutrient recovery into algal biomass, (3) the stability of species composition in the algal monoculture in outdoor open ponds, and (4) bacterial development in mixotrophic algal cultivation mode. The experiment used four 1 m^3^ algal ponds inoculated with the alga *Monoraphidium minutum* KAC90, two of which were run as a mixotrophic treatment using FGC‐cheese whey mixture, and the other two ponds as the photoautotrophic control treatment using FGC and potassium phosphates (KH_2_PO_4_). Measurements during the experiment included biomass loss, biomass weight, cell abundance, nutrient concentrations, biochemical composition of harvested biomass and eukaryotic/prokaryotic species composition. This study reveals insights into algal biomass dynamics, nutrient recovery, species stability and bacterial interactions associated with mixotrophic modes of algal cultivation, advancing sustainable biotechnological applications while addressing challenges associated with wastewater treatment and resource recovery.

## 2 Materials and Methods

### 2.1 Stock Culture

The alga *Monoraphidium minutum* KAC90, isolated in 2018 (Nham et al. 2023), has since been maintained in autoclaved seawater (salinity 7‰) supplemented with f/2 ingredients (Guillard 1975), at 16°C and 50 μmol photons m^−2^ s^−1^ in a 16 L:8D cycle (ALGOLAND project, Linnaeus University). The culture was inoculated to autoclaved tap water supplemented with f/2 ingredients (Guillard 1975), at the similar light and temperature conditions, for 1 month. To obtain a large volume of stock culture for inoculation into raceway ponds, 150 L of *M. minutum* culture was cultivated in the filtered tap water supplemented with f/2 ingredients in 20 L plastic bags at the similar light and temperature conditions. In addition, air was pumped through a filter of 0.2 μm pore size before entering the cultures for bubbling and supplying CO_2_ for the algae.

### 2.2 Wastewaters

Flue gas condensate water (FGC) was collected from Kalmar Energi power plant in October 2023. At this timepoint, the factory was operated at 10%–15% working capacity, resulting in a low NH_4_^+^ concentration in the FGC (total nitrogen (TN) = 8.3 mg L^−1^). Ammonium chloride was therefore added to the FGC to increase TN up to the typical range of FGC at full‐capacity operation (35–50 mg L^−1^). Six 1 m^3^ tanks of FGC were kept outside (8°–16°C) during the experiment. The FGC used for the experiment was taken at 30 cm from the bottom of the tanks to avoid flocculated particles.

Cheese whey (50 L) was collected from the cheese production water flow (Kalmar Dairy factory, Arla). Details on the water flow in Kalmar Dairy factory are presented in Nham et al. (2023). Nutrients were analyzed and the cheese whey was stored at −20°C to be used later for the whole experiment. When used, the frozen cheese whey was thawed and added into the algal ponds.

### 2.3 Experimental Setup

In October 2022, the algal strain *M. minutum* was inoculated into four outdoor raceway ponds (1 m^3^ each, RV3.4, MicroBio Engineering Inc.) containing a mixture of diluted FGC (3:7 ratio of FGC to tap water by volume) and KH_2_PO_4_ prepared according to the f/2 recipe (Guillard 1975). The working volume of the ponds was set at 20 cm depth of the water column. The culture in each pond was mixed by a paddle wheel at a speed of 15–30 cm s^−1^. Temperature, light, and pH sensors were installed to enable the control unit's continuous data recording (MicroBio Engineering Inc.). This unit also regulated the injection of flue gas stream (10%–15% CO_2_) through valves that opened at pH > 8 and closed at pH < 7. In‐culture temperature was automatically logged every 5 min, while light intensity was recorded at 20 cm above the culture surface at 15‐min intervals.

The experiment was preceded by two acclimatization phases. In the first phase, the cultures were maintained in FGC‐KH_2_PO_4_ medium for 8 days to acclimatize to the outdoor environmental conditions and the medium. In the second phase, lasting 6 days, two ponds were maintained in the same medium as before and defined as the photoautotrophic control treatment (PA). The other two ponds were supplemented with cheese whey instead of KH_2_PO_4_, as a source of P and OC, becoming the mixotrophic CW treatment. Notably, KH_2_PO_4_ or cheese whey was added to achieve an N:P mole ratio of 16 in the final media. Trace metals and vitamins were added according to the f/2 recipe (Guillard 1975) at the beginning of each cycle to ensure sufficient growth media for the algae.

After the acclimatization phases were completed, the experiment began when 30% culture volume was replaced by fresh medium according to the treatments described above. The replacement was repeated every second day for nutrient resupply. To accommodate comparable biomass concentrations (measured as optical density [OD]) between treatments at the beginning of each cycle, a 5% volume replacement was applied in the photoautotrophic ponds, while a 30%–40% volume replacement was used in the mixotrophic ponds. At each replacement, the cultures were harvested for biomass collection, which was later used for biochemical analysis. Each replacement marked the end of a cultivation cycle and the beginning of a new one, resulting in five cultivation cycles over ten experimental days. Cheese whey was added at 0.5% (cycle 1), 0.75% (cycle 2), 0.88% (cycle 3), 1% (cycle 4) and 1.25% (cycle 5) to reach N:P mole ratio of approximately 16 in the CW treatment. The initial TN concentrations in the CW (24.93 ± 6.97 mg L^−1^) were higher than those in the PA (16.80 ± 2.33 mg L^−1^), but in both treatments added in excess.

### 2.4 Molecular Identification of Eukaryotic and Prokaryotic Taxa

Biomass was collected at the beginning of each acclimatization phase (Day−14 and −6) and at the end of each cultivation cycle (Day 2, 4, 6, 8, and 10) by filtering 10 mL culture through a 47 mm 0.2 μm Supor membrane filter (Pall) and immediately stored at −80°C. DNA was extracted using the FastDNA spin kit for soil (MP Biomedicals) protocol with modifications as described in (Nham et al. 2023).

Amplification of 18S and 16S rRNA gene markers was conducted by PCR using 454F‐981R primers (Stoeck et al. 2010) and 341F‐805R primers (Herlemann et al. 2011), respectively. The thermal program for the former primer pair consisted of an initial denaturation at 98°C for 30 s; 20 cycles of denaturation at 98°C for 10 s, annealing at 57°C for 45 s and extension at 72°C for 12 s; and final extension at 72°C for 2 min. The thermal program for the later primer pair consisted of initial denaturation at 98°C for 30 s; 12 cycles of denaturation at 98°C for 10 s, annealing at 62°C for 30 s and extension at 72°C for 5 s; and final extension at 72°C for 2 min. The PCR product was cleaned using AMPure XP beads‐based reagent protocol for PCR purification (Beckman Coulter). The cleaned PCR product was quantity and quality checked by Qubit 4 and NanoDrop 2000 Spectrophotometer (ThermoFisher Scientific). The cleaned PCR product was later attached with indexes by another PCR round using the corresponding thermal program as reported above. The output was cleaned and checked for DNA quantity and quality following the above method. The cleaned index‐attached PCR amplicons were pooled together at equal concentrations and sent to National Genomic Infrastructure (NGI) for sequencing using the Illumina MiSeq platform.

Sequencing data were processed using the nf‐core/ampliseq pipeline ver. 2.3.2 (Nextflow 21.10.6) with Pr2 database for the 18S sequences and SILVA138 database for the 16S sequences. The pipeline was run on Uppmax (Uppsala Multidisciplinary Center for Advanced Computational Science). Result summary of the workflow is shown in Tables S1 and S2. The sequencing data were deposited in the Short Read Archive (SRA) of the National Center for Biotechnology Information (NCBI) with the accession number PRJNA1190867.

### 2.5 Nutrient Analyses

Nitrogen and phosphorus concentrations in the forms of ammonium (NH_4_^+^), nitrates (NO_3_^−^), nitrites (NO_2_^−^), TN, phosphates (PO_4_^3^^−^)/total phosphorus (TP), and chemical oxygen demand (COD) were analyzed by the Hach Lange kits LCK304 (0.015–2 mg L^−1^), LCK339 (0.23–13.5 mg L^−1^), LCK341 (0.015–0.6 mg L^−1^), LCK138 (1–16 mg L^−1^), LCK349 (0.05–1.5 mg L^−1^), and LCI400 (0–1000 mg L^−1^), respectively. The absorbance was measured using DR3900 spectrophotometer (Hach Lange).

### 2.6 Biomass Measurement

OD was measured at wavelength 750 nm using UV1600PC spectrophotometer. For dry weight (DW), 20 mL of culture was filtered through a preweighed GF/F filter (Whatman Ø 47 mm, 0.45 µm pore size), prepared by filtering through 10 mL of deionized water and drying at 100°C overnight. The filtered biomass was washed twice with deionized water, dried overnight at 100°C and weighed. Dry weight was calculated as the difference in weight between the pre‐weighted filter and the dried filter, divided by the filtered culture volume. Notably, the filters used for DW had 0.45 µm pore size, DW excluded most of suspended bacteria which have median diameter of 0.22–0.335 µm (Joint and Pomroy 1987). Therefore, DW was considered as algal biomass dry weight.

For cell abundance of *M. minutum*, 2 mL of culture was fixed with acid Lugol's solution and stored in cold (4°C) darkness. When analyzed, the samples were diluted 10 times and sonicated to disperse attached and aggregated cells with a setting of 60% capacity for 2 cycles (45 s per cycle) with a pause of 15 s in between (Vibracell, Sonics). Algal cells were counted using sedgewick‐rafter chambers and an inverted microscope (Olympus).

Bacterial cell abundance was measured by flow cytometry. Every day, 1 mL of culture was taken from each pond at sunrise and sunset and fixed with Grade I glutaraldehyde at 1% final concentration and stored at −80°C. Before analysis, the samples were thawed, diluted three times and sonicated following the setting described above for microscopic algal enumeration. Before the measurement, the samples were diluted 100 times, and stained with SYBR Green (Invitrogen) to a final concentration of 1:10 000 (Marie et al. 2005). The flow cytometer was set at a flow rate of 50 μL min^−1^, and 20660 ‐ 108741 events per sample were analyzed. Cells were identified using forward light scatter (FSC), side light scatter (SSC), and green fluorescence from SYBR Green (525 nm) and counted using the CytExpert software 2.4 (Beckman Coulter).

To assess the performance of the algal cultures, five parameters were considered. Algal growth rate ${µ}_{\text{algae}}$ (d^−1^) and algal biomass productivity (mg L^−1^ d^−1^), both based on dry weight at sunrise of a day and that of the following cultivation day (${\text{DW}}_{\mathrm{sr}1}$ and ${\text{DW}}_{\mathrm{sr}2}$) (Equations 1 and 2). Nutrient removal rate (mg L^−1^ d^−1^) was calculated by Equation 3, in which ${\text{removal rate}}_{x}$ is removal rate of species × (TN, TP, or COD), ${\text{conc}}_{1}(x)$ and ${\text{conc}}_{2}(x)$ are concentrations of species × at sunrise of a day and that of the following cultivation day. The time interval for Equations (1), (2), (3) was 1 (day).

(1) $${µ}_{\text{algae}}=\frac{\ln\left({\text{DW}}_{\mathrm{sr}2}/{\text{DW}}_{\mathrm{sr}1}\right)}{\mathrm{time interval}}$$

(2) $$\text{Algal biomass productivity}\;=\frac{{\text{DW}}_{\text{sr}2}-{\text{DW}}_{\text{sr}1}}{\mathrm{time interval}}$$

(3) $${\text{Nutrient removal rate}}_{x}=\frac{{c\text{onc}}_{1}(x)-{c\text{onc}}_{2}(x)}{\mathrm{time interval}}$$

(4) $$\text{Night biomass loss}\;=\frac{{\text{DW}}_{\mathrm{ss}1}-{\text{DW}}_{\text{sr}2}}{{\text{DW}}_{\mathrm{ss}1}}*100$$

(5) $$\text{Night biomass loss rate}\;=\frac{\ln\left({\text{DW}}_{\mathrm{ss}1}\right)-\ln\left({\text{DW}}_{\text{sr}2}\right)}{\Delta t}$$

Night biomass loss (%) was evaluated from dry weight at sunset of a day (${\text{DW}}_{\mathrm{ss}1}$) and that at sunrise of the following cultivation day (${\text{DW}}_{\text{sr}2}$) (Equation 4) (Borovkov et al. 2019). Adapted to the equation Equations 1 and 3, night biomass loss rate (× 10^−^^3 ^h^−1^) was estimated as decrease of the biomass divided by the night length Δt (hours) (Equation 5).

### 2.7 Analysis of Biochemical Composition in Algal Biomass

Biomass was dewatered by gravity sedimentation for 3–5 h in a cool room (4°C) and concentrated by Pall TFF filtration unit (0.45 µm pore size). Algal slurries were then washed twice by deionized water and centrifuged at 10,500 × *g* (Beckman Avanti j‐25 centrifuge) for 15 min to remove water and soluble salts before stored at −20°C. The frozen pellets were freeze dried until constant weight (SvanVac freeze dryer), grinded into fine grains, homogenized and then preserved in a desiccator for later biochemical analyses.

Lipids (% DW) were extracted using the Blight and Dyer method (Bligh and Dyer 1959) with modifications as outlined by Nham et al. (2024). Lipids were extracted three times by vortexing 100 mg biomass in chloroform‐methanol solution (2:1 v/v), followed by sonication and then overnight incubation in 4°C and darkness. After each extraction, the sample was centrifuged at 2800 × g for 10 min using Heraeus Megafuge 8. The supernatant, containing extracted lipids, was then mixed with 0.73% NaCl solution (final volume ratio of 8:4:3 chloroform:methanol:NaCl) to remove residual water. Following another centrifugation at the same speed and duration, the upper liquid was collected, filtered through a 0.2 μm polytetrafluoroethylene membrane filter (VWR International), and dried at 60°C until constant weight.

Proteins (% DW) were measured by the folin‐phenol protein measurement method (Lowry et al. 1951) using the Bio‐Rad DC Protein Assay. Carbohydrates (% DW) were analyzed by the phenol sulfuric acid method (Nham et al. 2023). The final solution was spectrophotometrically measured at wavelength 750 nm for proteins and 490 nm for carbohydrates using Hach Lange DR3900.

### 2.8 Statistical Analyses

All statistical analyses were performed using R version 4.2.0 with the significance level *p* < 0.05. Normality and homogeneity of the data on algal/bacterial growth rate and productivity, night biomass loss, and nutrient removal rate in the photoautotrophic and mixotrophic treatment were checked by the Shapiro‐Wilk test and Levene's test, respectively. For comparison of algal growth rate and productivity, bacterial growth rate, nutrient removal rate, and night biomass loss (rate) between the CW and PA treatment, t‐test was applied for normally distributed data and Wilcoxon test was used for non‐normal data.

## 3 Results

### 3.1 Cultivation Conditions

**Figure 1:**
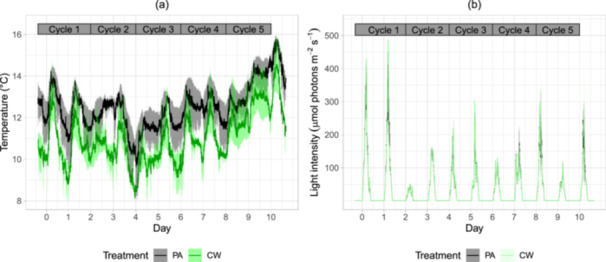
Temperature inside the algal ponds (a) and irradiance above the algal pond surface (b) of the photoautotrophic control (PA) and cheese whey mixotrophic treatment (CW) over the experimental time. The lines display average values for two ponds of each treatment (*n* = 2) and the surrounding areas of corresponding colors show standard deviation. Cultivation days are marked at the timepoint sunrise for each day.

The FGC contained N mainly as NH_4_^+^, and no P (Table 1). Therefore, an addition of P to FGC was required to make a full medium for algae, that is, KH_2_PO_4_ for the PA treatment and cheese whey (N:P mole ratio of 4.9) for the CW treatment. The cheese whey contained a high concentration of COD at 50,950 mg L^−1^, providing OC for mixotrophic growth in the CW treatment. During the experiment, temperature inside the ponds fluctuated from 7.8°C to 15.8°C, within the range for air temperature, 1.1°C–16.5°C (SMHI.se) and within the optimal range of adaptation for Nordic algae species (Cheregi et al. 2019). The differences in temperature between ponds ranged from 0°C to 4°C (Figure 1a). Although the maximum light intensity measured above the surface of the cultures fluctuated highly between 50 and 500 μmol photons m^−^^2^ s^−1^ over several days, it remained consistent across the four ponds (Figure 1b). The daylength ranged in 7–8 h, defined by our measured light intensity above the pond surface higher than 1.2 μmol photons m^−2^ s^−1^. That matches the time distance of 7–9 h of global irradiance > 5 W m^−2^ per day despite the sunshine time of 0–8 h per day during the experimental time (Figure S1).

**Table 1:** Characteristics of the flue gas condensate water and cheese whey water.

	Flue gas condensate water	Cheese whey water
pH	7.0	6.6
COD (mg L^−1^)	4	50,950
TN (mg L^−1^)	8.3	769
TP (mg L^−1^)	0	350
NH_4_^+^ ‐ N (mg L^−1^)	6.9	6.6
NO_2_^‐^ ‐ N (mg L^−1^)	0.7	0.2
NO_3_^‐^ ‐ N (mg L^−1^)	0.4	3.6
PO_4_^3‐^ ‐ P (mg L^−1^)	0	3

Abbreviations: COD, chemical oxygen demand; NH_4_^+^, ammonium; NO_2_^−^, nitrites; NO_3_^−^, nitrates; PO_4_^3^^−^, phosphates; TN, total nitrogen; TP, total phosphorus.

**Figure 2:**
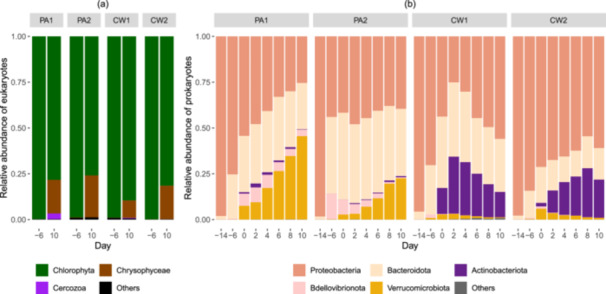
18S and 16S rRNA gene amplicon‐based relative abundance of eukaryotes (a) and prokaryotes (b) in the four algal ponds of photoautotrophic control (ponds PA1 and PA2) and cheese whey treatment (ponds CW1 and CW2) at different timepoints. The plots show relative abundance in % of all phyla in the ponds at Day −14 (at inoculation of the stock culture of *Monoraphidium minutum* KAC90 from the laboratory into the outdoor ponds); Day −6 (before adding cheese whey into the CW treatment ponds for acclimatization); Day 0 (the acclimatization period finished, all samplings were started); and Days 2–10 (at the end of cultivation cycles 1–5).

At start (Day −14), a nonaxenic monoculture of *M. minutum* was inoculated into all four raceway ponds. After 8 days of acclimatization in FGC water (Day −6), the eukaryotic community was dominated by phylum Chlorophyta, 98.8%–99.9% (Figure 2a). At the end of the experiment (Day 10), Chlorophyta remained the dominant phylum in both treatments, although its relative abundance decreased to 75.9%–81.3%. Within the phylum Chlorophyta, the genus *Monoraphidium* was the most dominant throughout the experimental time, ranging from 99.8% to 100%. At amplicon sequence variants (ASVs) level, *M. minutum* was the most abundant genus with two ASVs whose total abundance in all samples was 75.2%–99.6% total reads (Figure S2). Besides Chlorophyta, Chrysophyceae increased its relative abundance from nonsignificant on Day −6 to 9.4%–22.9% by Day 10 in both treatments (Figure 2a). This phylum was dominated by the genus *Spumella*, containing 3 dominating ASVs representing 99.3%–100% of total Chrysophyceae (Figure S2).

The prokaryotic community was initially dominated by phylum Proteobacteria, with a relative abundance of 97.4% at inoculation into the outdoor ponds (Day −14) (Figure 2b). Later (from Day −6 to Day 10), Proteobacteria (relative abundance of 25.1%–84.3%) and Bacteroidota (14%–47.4%) were the most abundant phyla in both treatments. Additionally, the PA control showed an increased abundance of Verrucomicrobiota from negligible on Days −14 and −6 to 2.7%–45.4% in Days 0–10, while the CW treatment had an increased abundance of Actinobacteriota, reaching 2%–31.1% in Days 0–10. Additionally, Bdellovibrionota became relatively abundant in PA (0.6%–8.1%) but was minor in CW treatment (0%–1.2%) in Days 0–10. In summary, Proteobacteria and Bacteroidota were the most dominant over the experimental time, followed by Verrucomicrobiota and Bdellovibrionota in the PA control, and Actinobacteriota in the CW treatment (Figure 2b).

### 3.2 Biomass Growth and Night Biomass Loss

**Figure 3:**
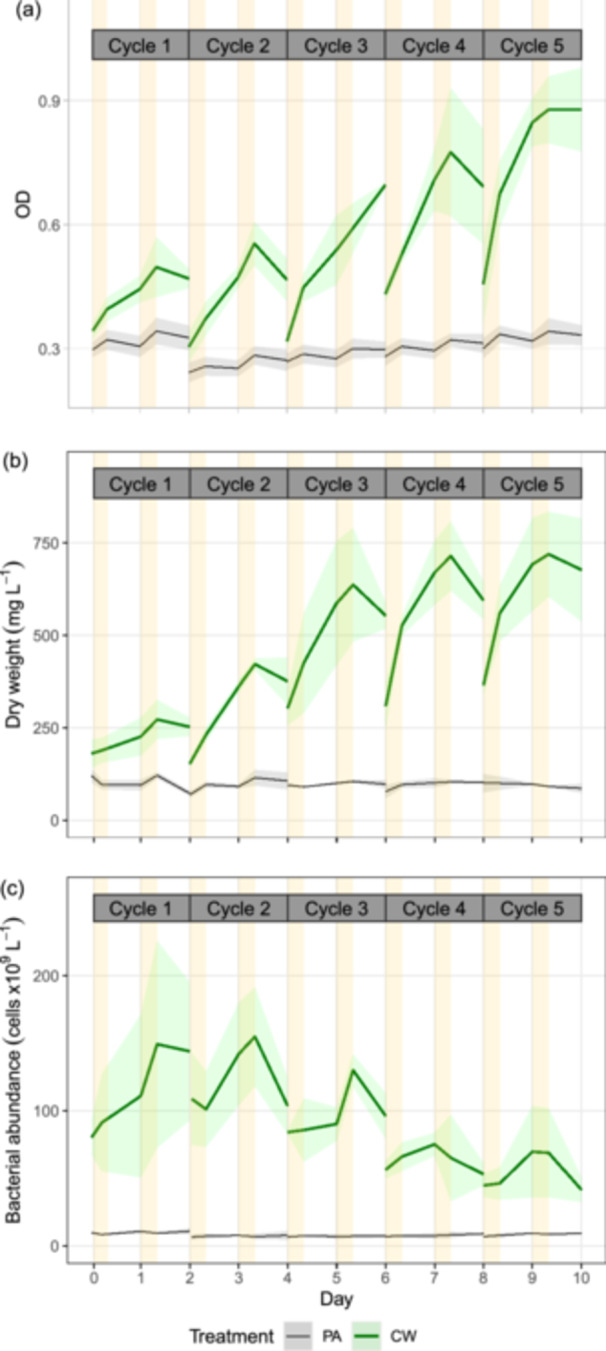
Biomass growth in photoautotrophic control (PA) and cheese whey mixotrophic treatment (CW), shown by OD (a); dry weight (b); and bacterial abundance (c) after the acclimatization period. The lines show average values and the shadow areas of corresponding color show standard deviation (*n* = 2). Yellow areas show daytime period when sunlight is present. Cultivation days are marked at the timepoint sunrise for each day.

Total biomass mainly consisted of eukaryotic algae (predominantly *Monoraphidium minutum* and *Spumella*) and heterotrophic bacteria in both PA and CW treatments (Figure 2 and Figure S2). Heterotrophic bacteria were more abundant in the CW than in the PA treatment (Figure 3c). No correlation between heterotrophic bacterial biomass and DW was found (Pearson, *p* > 0.05). In general, bacterial contribution to total biomass was negligible as on average, it was equal to 0.1% of *M. minutum*'s biomass in the PA and 1.1% of *M. minutum*'s biomass in the CW treatment (Figure S5). Thus, algae are mainly responsible for the growth of total biomass.

We used DW, instead of OD or cell count of *M. minutum*, for estimating algal biomass as algal biomass and algal productivity (based on DW) were positively correlated to nutrient removal rate (Pearson, *R* = 0.82–0.91, *p* < 0.05; Figure S3). Meanwhile, a poor correlation between OD and DW was observed in the PA (Pearson, *R* = 0.37, *p* < 0.01) (Figure S4). The decrease of biomass overnight in the PA was more pronounced on the OD graph than on the DW graph (Figure 3a,b), agreeing with Edmundson and Huesemann (2015) reporting that an use of OD for calculation night biomass loss can result in an overestimation compared to the use of DW. Also, the biomass of *M. minutum* (estimated based on microscopic cell count) was poorly correlated with DW (Figure S5, Pearson: *p* > 0.05 for both PA and CW treatment).

**Figure 4:**
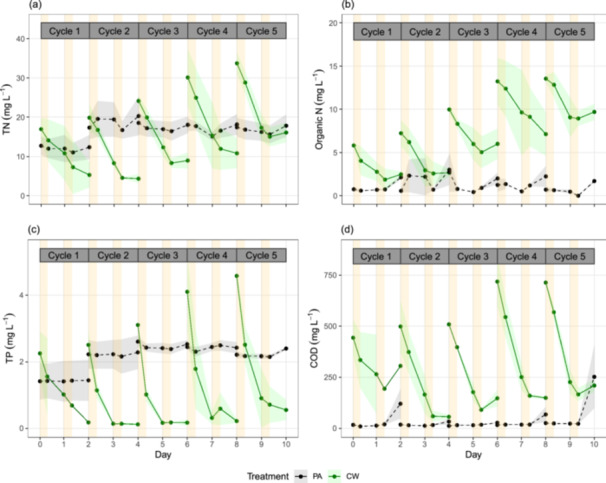
Changes of dissolved nutrients (mg L^−1^) as TN (a), organic N (b), TP (c), and COD (d) in the photoautotrophic (PA) and cheese whey mixotrophic (CW) treatments. The lines show average values and the shadow areas of corresponding color show standard deviation (*n* = 2). Cultivation days are marked at the timepoint sunrise for each day. COD, chemical oxygen demand; Organic N, organic nitrogen; TN, total nitrogen; TP, total phosphorus.

The algae in the CW treatment grew exponentially on the first days of all cycles and entered the stationary phase on the second days of all cycles (Figure 3b), which was consistent with its higher nutrient removal on the first days compared to the second days of all cycles (Figure 4). On the first days of all cycles, the CW treatment showed significantly higher growth rate, productivity, and TN/TP/COD removal rates than the PA control (*t*‐test, *p* < 0.05) (Table 2), suggesting that OC in cheese whey promoted mixotrophic growth of the algae. On the second days of all cycles, however, the CW treatment had equal productivity, but higher nutrient removal rate (TN, TP, COD) compared to the PA control (Table 2).

**Table 2:** Algal growth rate; algal productivity; and removal rate of total nitrogen (TN), total phosphorus (TP), and chemical oxygen demand (COD). Day 1 of cycles represent the time intervals from the start of each cycle (sunrise timepoint) to the sunrise of the next day. Day 2 of cycles last from the sunrise of Day 2 to the end of each cycle (24h later). Mean values are calculated as average of five cycles in each treatment (*n* = 10). Significant difference in each parameter per day between photoautotrophic and mixotrophic is shown by an asterisk (*p* < 0.05) or two asterisks (*p* < 0.01) or three asterisks (*p* < 0.001). Nonsignificant difference is shown by “ns” abbreviation.

Time	Parameter	PA	CW	Sig. difference	Statistical test
Day 1 of cycles	Algal growth rate (d^−^^1^)	0.07 ± 0.23	0.63 ± 0.27	***	*t*‐test
	Algal productivity (mg L^−1^ d^‐1^)	4.75 ± 21.33	244.95 ± 138.42	***	*t*‐test
	TN removal rate (mg L^−1^ d^−1^)	0.88 ± 2.09	12.13 ± 4.06	***	*t*‐test
	TP removal rate (mg L^−1^ d^−1^)	0.05 ± 0.09	2.80 ± 1.08	***	*t*‐test
	COD removal rate (mg L^−1^ d^−1^)	1 ± 5	359 ± 127	***	*t*‐test
Day 2 of cycles	Algal productivity (mg L^−1^ d^−1^)	−4.00 ± 16.04	−16.75 ± 62.31	ns	*t*‐test
	TN removal rate (mg L^−1^ d^−1^)	−1.39 ± 1.60	3.71 ± 2.99	**	Wilcoxon
	TP removal rate (mg L^−1^ d^−1^)	−0.08 ± 0.10	0.26 ± 0.55	*	Wilcoxon
	COD removal rate (mg L^−1^ d^−1^)	−84 ± 102	44 ± 113	**	Wilcoxon

**Figure 5:**
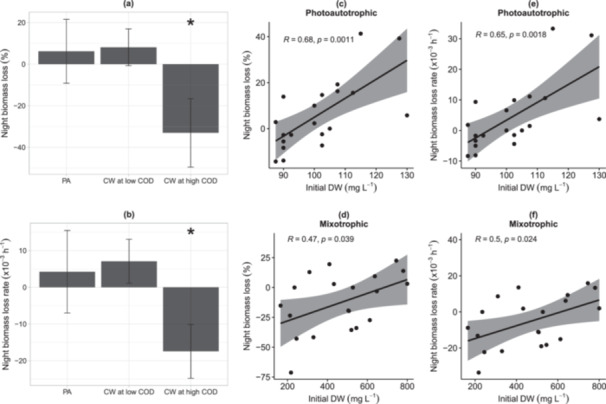
Night biomass loss (%) (a), night biomass loss rate ( × 10^−3^ h^−1^) (b), and their correlation with initial biomass concentration in dry weight (DW) (c–f). Night biomass loss (a) or night biomass loss rate (b) was displayed as the average of all five cycles in the photoautotrophic control (PA) (*n* = 20 ± SD); and cheese whey (CW) mixotrophic cultures at low organic C (36 ‐ 189 mg L^−1^ COD) (*n* = 10 ± SD) and high organic C (239–613 mg L^−1^ COD) (*n* = 10 ± SD). Significant differences between treatments (*) (*p* < 0.05) (*t*‐test). The correlation plots indicate regression line and the shadow areas present confidence intervals (*n* = 20) (c–f).

On the first nights of all cycles, with the initial COD at sunset (onset of the nights) ranging 239–613 mg L^−1^, night biomass loss and night biomass loss rate were negative (Figure 5a,b). That was consistent with a night gain on average 33% of the biomass synthesized during daytime in the CW treatment. However, on the second nights of all cycles (initial COD of 36–189 mg L^−1^ at sunset), the average night biomass loss was 8%, which was not significantly different from the PA treatment's average of 6% (*t*‐test, *p* > 0.05) (Figure 5a). Similarly, the night biomass loss rate, ranging from 33.3 to −8.4 (× 10^−3 ^h^−1^), did not differ between PA and CW treatment when the initial COD was low (Figure 5b). Also, night biomass loss and night biomass loss rate were positively correlated with initial biomass concentration (DW) in both the PA and CW treatment (Pearson, *R* = 0.47–0.68, *p* < 0.05) (Figure 5c–f). In other words, night biomass loss and loss rate were higher in denser cultures.

Algal night metabolism depends on numerous environmental factors, including nutrient status in medium, temperature, the prior light intensity history, and algal species itself (Le Borgne and Pruvost 2013). During the experiment, initial concentrations of COD, TN and TP at the onset of night 1 of cycles were higher than those of night 2 of cycles (Figure S6). To reveal the correlation between nutrient concentration in medium and night biomass loss, we ran a multiple linear regression model estimating night biomass loss depending on initial concentrations of COD, TN and TP at the onset of darkness (Table S3). The result showed that initial TN and TP concentrations were not significant in the multiple regression model (*p* > 0.05), meaning these parameters did not significantly affect the night biomass loss. In other words, the cultures did not suffer nitrogen or phosphorus stress although their dissolved concentrations in medium were lower at night 2 compared to night 1 of cycles. However, initial COD had a significant effect on night biomass loss. A linear regression between night biomass loss and initial COD showed that an increase of initial COD by 100 mg L^−1^ could lead to an expected decrease of 9.76% in night biomass loss on average (*p* = 0.000, coefficient = −0.0976, intercept = 16.7047, Table S3). It also means that an initial COD was required to be over 171 mg L^−1^ on average to prevent night biomass loss.

### 3.3 Biochemical Composition of Algal Biomass

**Figure 6:**
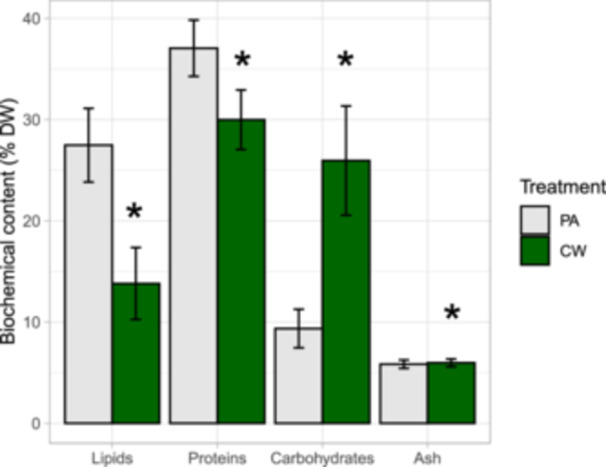
Biochemical composition (% dry weight) in algal biomass from photoautotrophic (PA) and cheese whey mixotrophic treatments (CW) (*n* = 10 ± SD) (two ponds per treatment within five cycles). Significant differences in all biochemical compositions—lipids (Wilcoxon), proteins, (Wilcoxon) carbohydrates (*t*‐test), and ash content (*t*‐test)—were observed between treatments (*) (*p* < 0.05).

Biochemical composition in algal biomass was shown as lipid, protein, carbohydrate and ash content (Figure 6). The CW treatment had an average lipid content of 13.8% DW and a protein content of 30% DW, which was significantly lower than in the PA control (27.5% and 37.1% DW, respectively) (Wilcoxon, *p* < 0.05). Meanwhile, the average carbohydrate content in the CW treatment (26% DW) was higher than in the PA (9.4% DW) (*t*‐test, *p* < 0.05). Ash content of the biomass is visually similar between treatments (Figure 6), despite a significant difference (*t*‐test, *p* < 0.05).

## 4 Discussions

### 4.1 Biomass Growth and Night Biomass Loss

Total biomass was mainly contributed by algae, with *M. minutum* being the dominate species. However, a poor correlation between cell count–based biomass of *M. minutum* and algal DW could be caused by changes of cell size/weight and the decrease of this strain's relative abundance from 99.6% total reads at day −6 down to 75.2% total read (18S rRNA data) at day 10. In open cultivation systems, such a biocontamination is unavoidable over time. Here, *Spumella* exhibited as a good adaptive species to the cultivation conditions and increased its relative abundance during the experiment. Since the aim of our cultivations are high biomass yield, *Spumella* had minor effect since the slope of DW on the first day of cycles did not decrease over time. However, biocontamination needs to be monitored if the cultivation is kept longer.

Algal biomass production was significantly enhanced in the CW treatment, which showed higher growth rate, productivity and nutrient removal rate compared to the PA control on the first days of all cycles. This observation is consistent with previous studies reporting better biomass gain and nutrient removal in mixotrophic cultures than photoautotrophic cultures (Gupta et al. 2016; Lari and Khosravitabar 2021; Nham et al. 2024). During the first night across all cycles (initial COD = 239–613 mg L^−1^), CW treatment increased biomass concentration by an average of 33%, indicating algal heterotrophic growth and a prevention of night biomass loss. The result is consistent with others reports showing a reduction or prevention of night biomass loss when OC was added to algal cultures at a laboratory scale (Nair and Chakraborty 2020; Ogbonna and Tanaka 1996, 1998).

On average, night biomass loss was prevented when initial COD ≥ 171 mg L^−1^ at the onset of darkness. As night biomass loss is caused by respiration and cell mortality (Le Borgne and Pruvost 2013), the minimal amount of COD was required for cell maintenance and heterotrophic growth compensating for cell death. Therefore, night biomass loss in the CW treatment (8%) on the second nights of all cycles (initial COD = 36–189 mg L^−1^), was comparable to that in the PA control (6%). These night biomass loss values falls within the typical range for photoautotrophic cultures (3%–8%) (Abiusi et al. 2020). This night biomass loss contributed to the equal algal productivity observed between treatments on the second day of cycles. However, the nutrient removal rate in the CW treatment was higher than in the PA control on the second day, suggesting that nutrients were absorbed for mixotrophic biomass growth during daytime and heterotrophic growth at night. Additionally, nutrient removal was occasionally negative during the second night of cycles in both treatments (Figure 4), which agrees with a previous report on nutrient release at low COD in low light conditions (Nham et al. 2024).

Night biomass loss in each treatment showed high variations between cycles (Figure 5a). This is likely because night algal biomass losses depend on the temperature at night and daytime light intensity (Gaidarenko et al. 2019), which changed hourly and daily during the experiment. Additionally, our study aligns with other studies that reported a positive correlation between night biomass loss and biomass concentration, as high biomass concentrations entail higher costs for cellular maintenance during the night (Holdmann et al. 2018; Michels et al. 2014).

Changes of night biomass loss rate in photoautotrophic cultures against biomass concentration exhibit a unimodal relationship, with positive correlations at low cell densities and negative correlation at very high cell densities (Holdmann et al. 2018; Michels et al. 2014). The positive relationship between night biomass loss rate and DW in our study indicates that our cell concentrations fall within the low cell density range. This is in agreement with de Vree et al. (2016), who reported an increase of night biomass loss rate at low biomass concentration in photoautotrophic cultures. Therefore, at certain cultured cell concentrations, adjustments of the harvest frequency can reduce night biomass loss.

### 4.2 Biochemical Composition of Algal Biomass

Biochemical composition of the biomass in the PA treatment (proteins: 37 ± 3% DW, carbohydrates: 9% ± 2% DW, lipids: 27% ± 4% DW) was comparable to a previous lab‐scale cultivation of the dominant algal strain *M. minutum* KAC90 (Nham et al. 2023). Meanwhile, protein content in the CW treatment (30% ± 3% DW) was lower than in the PA treatment, which is in agreement with previous studies reporting that overnight protein content increased in photoautotrophic cultures and decreased in mixotrophic cultures on glucose (Abiusi et al. 2020; Ogbonna and Tanaka 1996, 1998). Carbohydrate content in the CW treatment (26% ± 5% DW) was significantly higher than in the PA treatment, potentially because the algae consumed intracellular carbohydrates for cell maintenance and protein synthesis in the PA treatment (Ogbonna and Tanaka 1996), but they might use the added OC for cell maintenance and division in the CW treatment. Additionally, lipid content in the CW treatment (14 ± 4% DW) was significantly lower than in the PA treatment, which is consistent with previous findings that heterotrophic biomass of *Monoraphidium* had lower lipid content compared to photoautotrophic one (Yu et al. 2012).

Ash content in the CW and PA treatment (5.7%–6.1% DW) was close to the reported ash content for *Monoraphidium*, 6.6%–10.4% DW (Singh et al. 2023; Ekendahl et al. 2018). The cheese whey medium could possibly contain slightly higher concentrations of ions, for example, K^+^ and Ca^2+^, than in the PA as these ions are abundant in milk (Pereira 2014). Some algal species were reported to accumulate those ions in seawater medium (Szelag‐Sikora et al. 2016). Thus, the slight difference in ash content between the CW and PA treatment could be caused by different species composition between treatments and possible accumulation of ions from cheese whey.

Concentration of TN and TP were quite low in the CW treatment at the end of cycles when biomass was collected for biochemical analysis. Nutrient stress can enhance lipid content in algal biomass (Maltsev et al. 2023). However, no correlation between night biomass loss and TN/TP suggests a sufficiency of N and P for algal metabolism during the second night of cycles, the time before harvesting. Also, there might be a delay in response of algae to the low nutrient levels in the medium. In conclusion, the result showed no notable effects of nutrient stress on the lipid content in algal biomass in the mixotrophic treatment.

### 4.3 Microbial Composition in Relation to Night Algal Biomass Loss

Algal monocultures are often thought to be unstable due to risks of invading species thriving in unoccupied niches (Padmaperuma et al. 2018) and therefore the selected species for monocultures must be robust in the used medium and prevailing cultivation conditions. The local strain *M. minutum* KAC90 dominated the eukaryotic community throughout the experimental time (day −6 to 10). Its robustness coincides with that of another *Monoraphidium* strain, growing vigorously in a multi‐species culture in a raceway pond during a cold season (Gao et al. 2023). Thus, in both PA and CW treatments, no matter how significant the night algal biomass loss was, *M. minutum* KAC90 maintained its domination in the cultures.

Proteobacteria and Bacteroidota were the dominant prokaryotic phyla in both PA and CW treatment. These phyla are often found to dominate the noncyanobacterial prokaryotic communities associated with green algae in various algal applications (Ayre et al. 2021; Cai et al. 2014; Krohn‐Molt et al. 2013; Ramanan et al. 2015; Mohd Udaiyappan et al. 2020; Yang et al. 2023; Zhang et al. 2022; Zheng et al. 2022). Other highly abundant phyla were Actinobacteroida in the CW treatment and Verrucomicrobiota in the PA treatment, consistent with the previous reports showing them as two major partners with green algal and cyanobacterial cultivations (Ayre et al. 2021; Cai et al. 2014). Actinobacteroida are usually associated with high abundance of complex carbohydrates (Llamas et al. 2021), suggesting that these bacteria could be enhanced by carbohydrates in the CW treatment. In addition, Bdellovibrionota is a phylum of parasitic bacteria that feed on other bacteria (Yang et al. 2023). Over time, Bdellovibrionota became more abundant in the PA treatment, which concurs with the finding by Yang et al. (2023). However, this phylum was not dominant in the CW cultures, suggesting they were not as effective competitors as the other dominant phyla in the presence of cheese whey. Furthermore, the higher abundance of Bdellovibrionota could be associated with the low bacterial abundance in the PA treatment. In summary, it is possible that the lower relative abundance of Actinobacteroida and higher relative abundance of Verrucomicrobiota/Bdellovibrionota might play an important role in the high night algal biomass loss in the photoautotrophic cultures.

### 4.4 Potential of Flue Gas Condensate and Cheese Whey for Algal Cultivation

This study showed higher biomass productivity and nutrient removal in the CW treatment than in PA the control, which aligned with Patidar et al. (2014) indicating an enhanced biomass production of *M. minutum* in mixotrophic mode. We also identified cheese whey as a potential source of P and OC for outdoor large‐scale mixotrophic algal cultivation. Furthermore, streams of FGC contain significant amounts of heavy metals, salts and solids, which can negatively affect algae (Noor et al. 2021). The used FGC was collected when the factory was not working at full capacity, but it still contained heavy metals at levels below threshold values for discharge (Kalmar Energi environmental report). However, the result showed growth rates of 0.63 ± 0.27 d^−1^ in the CW treatment on the first days of all cycles, close to that of *M. minutum* KAC90 growing photoautotrophically under optimal light and temperature conditions (Nham et al. 2023). This finding suggests that the toxic effects of substances present in FGC are minimal and supports the conclusion that an FGC‐cheese whey mixture provides a viable medium for mixotrophic algal cultivation. In addition, since the NH_4_^+^ concentration in the FGC varies depending on the factory operations, it is necessary to adjust the mixing ratio with cheese whey to make a nutrient‐balanced medium for algae.

## 5 Conclusions

Nordic autumn conditions, characterized by low temperatures and limited light (both in intensity and daylength), are not optimal for algal growth. Despite these challenges, *M. minutum* KAC90 was successfully cultivated in the mixture of flue gas condensate and cheese whey in pilot‐scale outdoor raceway ponds. The strain dominated eukaryotic community during the experiment as the ASVs of KAC90 comprised 75.2%–99.6% total reads in the 18S rRNA data. Cheese whey proved to be an excellent source of organic carbon for mixotrophic algae, enhancing algal growth and nutrient removal compared to the control treatment. Adequate addition of cheese whey in algal culture prevented night biomass loss at the tested algal biomass density levels. Algal biomass cultured in cheese whey was rich in carbohydrates, making it suitable for production of bioethanol and bioplastics.

## Author Contributions

Quyen Nham, Catherine Legrand and Elin Lindehoff conceived and designed the study. Quyen Nham, Tristan Gordon and Elin Lindehoff performed the experiments and wet lab analyses. Quyen Nham, Hanna Farnelid and Elin Lindehoff performed the data analyses. Quyen Nham and Elin Lindehoff wrote the original manuscript draft. Quyen Nham, Tristan Gordon; Hanna Farnelid, Catherine Legrand and Elin Lindehoff revised and edited the manuscript.

## Conflicts of Interest

The authors declare no conflicts of interest.

## Supporting information

MS6 v9 Supplementary QN.

## Data Availability

The data that support the findings of this study are available from the corresponding author upon reasonable request.
