# Supplementary material for: Mitigating Night Biomass Loss in Outdoor Pilot‐Scale Mixotrophic Algal Cultivation of Monoraphidium minutum Using Flue Gas Condensate and Cheese Whey
Source: Biotechnol Bioeng. 2025 Jul 18;122(10):2688–700. doi: 10.1002/bit.70027 (PMC12417766; doi:10.1002/bit.70027)
Supplement: Supplementary file 1 — MS6 v9 Supplementary QN. [file BIT-122-2688-s001.docx]

**Supplementary information**

Table S1. Overall summary of 18S rRNA gene sequence analysis

| No | Cultivation day | Pond | cutadapt total processed | cutadapt passing filters (%) | DADA2 input | filtered (%) | denoisedF (%) | denoisedR (%) | merged (%) | nonchim (%) |
| --- | --- | --- | --- | --- | --- | --- | --- | --- | --- | --- |
| 1 | -6 | CW1 | 322583 | 99.1% | 319769 | 80.4 | 80.4 | 80.4 | 80.2 | 79.1 |
| 2 | -6 | PA1 | 221588 | 99.3% | 219943 | 81.1 | 81.1 | 81.1 | 80.9 | 80.8 |
| 3 | -6 | CW2 | 239241 | 99.2% | 237345 | 81.1 | 81.1 | 81.1 | 80.9 | 80.7 |
| 4 | -6 | PA2 | 246696 | 99.1% | 244487 | 79.8 | 79.7 | 79.7 | 79.6 | 79.0 |
| 5 | 10 | CW1 | 214607 | 98.0% | 210421 | 79.6 | 79.5 | 79.5 | 79.1 | 71.2 |
| 6 | 10 | PA1 | 210138 | 99.2% | 208559 | 81.4 | 81.3 | 81.3 | 80.8 | 67.5 |
| 7 | 10 | CW2 | 180240 | 99.2% | 178875 | 81.4 | 81.3 | 81.3 | 80.7 | 68.7 |
| 8 | 10 | PA2 | 176202 | 98.8% | 174082 | 80.2 | 80.2 | 80.1 | 80.0 | 67.7 |

Table S2. Overall summary of 16S rRNA gene sequence analysis

| No | Cultivation day | Pond | cutadapt total processed | cutadapt passing filters (%) | DADA2 input | filtered (%) | denoisedF (%) | denoisedR (%) | merged (%) | nonchim (%) |
| --- | --- | --- | --- | --- | --- | --- | --- | --- | --- | --- |
| 1 | -14 | CW1 | 374111 | 95.9% | 358878 | 73.0 | 72.6 | 72.8 | 70.5 | 52.4 |
| 2 | -14 | PA1 | 329843 | 96.1% | 316862 | 73.0 | 72.7 | 72.8 | 71.0 | 54.3 |
| 3 | -14 | CW2 | 309321 | 96.0% | 296986 | 73.2 | 72.9 | 73.0 | 71.2 | 55.4 |
| 4 | -14 | PA2 | 269774 | 95.9% | 258732 | 72.0 | 71.8 | 71.9 | 70.2 | 55.3 |
| 5 | -6 | CW1 | 244004 | 95.9% | 234053 | 74.8 | 74.0 | 74.5 | 71.2 | 52.3 |
| 6 | -6 | PA1 | 249537 | 96.1% | 239877 | 75.9 | 75.0 | 75.5 | 72.4 | 52.3 |
| 7 | -6 | CW2 | 221560 | 95.9% | 212390 | 73.7 | 73.0 | 73.4 | 71.1 | 55.5 |
| 8 | -6 | PA2 | 191559 | 95.9% | 183744 | 74.2 | 73.3 | 73.8 | 71.0 | 55.6 |
| 9 | 0 | CW1 | 182372 | 96.1% | 175294 | 74.6 | 72.8 | 73.7 | 68.0 | 44.8 |
| 10 | 0 | PA1 | 178496 | 95.9% | 171242 | 75.6 | 74.1 | 75.0 | 70.3 | 55.9 |
| 11 | 0 | CW2 | 200670 | 96.1% | 192891 | 77.0 | 75.9 | 76.5 | 73.1 | 58.5 |
| 12 | 0 | PA2 | 169030 | 95.8% | 161935 | 75.2 | 74.2 | 74.7 | 71.2 | 59.5 |
| 13 | 2 | CW1 | 187505 | 96.0% | 180092 | 73.2 | 71.8 | 72.5 | 68.3 | 50.8 |
| 14 | 2 | PA1 | 172507 | 95.8% | 165256 | 75.3 | 73.8 | 74.7 | 70.5 | 59.4 |
| 15 | 2 | CW2 | 172810 | 96.0% | 165862 | 75.9 | 74.8 | 75.4 | 72.2 | 58.4 |
| 16 | 2 | PA2 | 167120 | 95.9% | 160275 | 75.9 | 74.5 | 75.3 | 70.6 | 56.7 |
| 17 | 4 | CW1 | 54813 | 96.2% | 52744 | 74.8 | 72.6 | 73.4 | 67.7 | 50.8 |
| 18 | 4 | PA1 | 145492 | 96.0% | 139679 | 75.8 | 73.8 | 75.1 | 69.4 | 55.0 |
| 19 | 4 | CW2 | 153045 | 96.2% | 147222 | 75.8 | 74.2 | 75.2 | 70.6 | 49.2 |
| 20 | 4 | PA2 | 125943 | 95.9% | 120761 | 76.2 | 74.3 | 75.3 | 69.6 | 54.8 |
| 21 | 6 | CW1 | 128633 | 96.1% | 123663 | 74.1 | 72.1 | 73.1 | 66.7 | 38.8 |
| 22 | 6 | PA1 | 140882 | 96.0% | 135207 | 75.4 | 73.5 | 74.6 | 69.7 | 56.8 |
| 23 | 6 | CW2 | 125435 | 96.0% | 120444 | 74.6 | 73.0 | 73.9 | 69.2 | 48.4 |
| 24 | 6 | PA2 | 128246 | 95.8% | 122855 | 76.5 | 75.5 | 76.1 | 73.4 | 68.7 |
| 25 | 8 | CW1 | 101762 | 96.0% | 97649 | 72.2 | 69.7 | 70.9 | 63.9 | 37.8 |
| 26 | 8 | PA1 | 110065 | 95.7% | 105376 | 72.6 | 70.4 | 71.7 | 66.8 | 58.3 |
| 27 | 8 | CW2 | 98568 | 94.7% | 93339 | 72.7 | 71.2 | 71.9 | 67.6 | 51.5 |
| 28 | 8 | PA2 | 96372 | 94.8% | 91319 | 73.4 | 71.6 | 72.6 | 67.7 | 57.3 |
| 29 | 10 | CW1 | 98167 | 95.8% | 94021 | 72.4 | 70.3 | 71.3 | 65.3 | 39.9 |
| 30 | 10 | PA1 | 104835 | 95.7% | 100319 | 71.7 | 69.9 | 71.0 | 66.7 | 59.1 |
| 31 | 10 | CW2 | 101098 | 95.9% | 96914 | 73.5 | 72.1 | 72.6 | 68.0 | 52.1 |
| 32 | 10 | PA2 | 92073 | 95.7% | 88116 | 73.7 | 71.5 | 72.8 | 66.9 | 55.9 |

Fig. S1. Sunshine time per day during the experimental days 0 - 10 (SMHI.se)

Fig. S2. 18S rRNA gene-based relative abundance of dominant ASVs (> 1% of total number of reads) in the photoautotrophic ponds (PA1, PA2) and mixotrophic ponds on cheese whey (CW1, CW2).

Fig. S3. Correlation between algal productivity and removal rates of total nitrogen (TN) removal rate (n = 40) (a), of total phosphorus (TP) removal rate (n = 40) (b), and of COD (n = 20) (c). The lines and shadow areas show regression lines and confidence intervals.


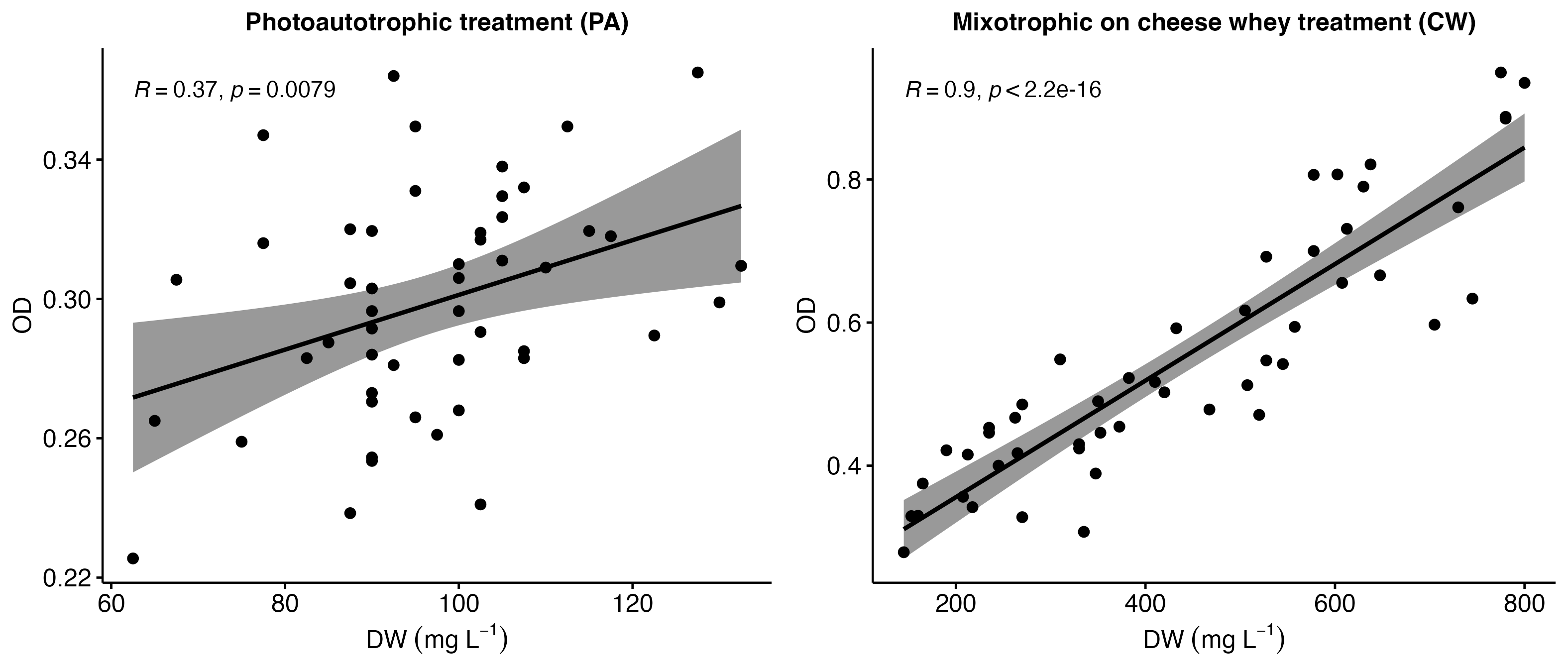


Fig. S4. Correlation between algal dry weight (DW) and optical density (OD) in the photoautotrophic (PA) and mixotrophic on cheese whey (CW) treatments. The lines and shadow areas show regression lines and confidence intervals (n = 50).

|  |
| --- |
|  |

Fig. S5. Development of algal dry weight, algal biomass of *Monoraphidium minutum* KAC90, and bacterial biomass in photoautotrophic treatment (a) and cheese whey treatment (b) (n = 2 ± SD). Algal biomass was estimated by multiplication of microscopic cell count with factor 5000 fg C per cell. Bacterial biomass was estimated by multiplication of cell count using flow cytometry with factor 33.1 fg C per cell. Data on algal biomass on day 10 was not available.

Fig. S6. Correlated night biomass loss with initial concentration of COD, TN (total nitrogen), and TP (total phosphorus) in the two ponds of the cheese whey treatments (CW1, CW2).

Table S3. Multiple linear regression of night algal biomass loss on initial concentration of COD, TN (total nitrogen), and TP (total phosphorus)

| > model <- lm(Loss ~ COD_initial + TN_initial + TP_initial, data = dt3)  > summary(model)  Call:  lm(formula = Loss ~ COD_initial + TN_initial + TP_initial, data = dt3)  Residuals:  Min 1Q Median 3Q Max  -40.412 -8.158 0.219 14.751 23.400  Coefficients:  Estimate Std. Error t value Pr(>\|t\|)  (Intercept) 17.97110 9.94924 1.806 0.0897 .  COD_initial -0.14550 0.05677 -2.563 0.0209 *  TN_initial 0.03587 1.44799 0.025 0.9805  TP_initial 11.64471 11.32484 1.028 0.3191  ---  Signif. codes: 0 ‘***’ 0.001 ‘**’ 0.01 ‘*’ 0.05 ‘.’ 0.1 ‘ ’ 1  Residual standard error: 18.41 on 16 degrees of freedom  Multiple R-squared: 0.5637, Adjusted R-squared: 0.4819  F-statistic: 6.891 on 3 and 16 DF, p-value: 0.003427 |
| --- |
| > model3 <- lm(Loss ~ COD_initial, data = dt3)  > summary(model3)  Call:  lm(formula = Loss ~ COD_initial, data = dt3)  Residuals:  Min 1Q Median 3Q Max  -44.534 -9.372 7.894 11.398 21.870  Coefficients:  Estimate Std. Error t value Pr(>\|t\|)  (Intercept) 16.70472 7.51230 2.224 0.039213 *  COD_initial -0.09761 0.02189 -4.458 0.000304 ***  ---  Signif. codes: 0 ‘***’ 0.001 ‘**’ 0.01 ‘*’ 0.05 ‘.’ 0.1 ‘ ’ 1  Residual standard error: 18.11 on 18 degrees of freedom  Multiple R-squared: 0.5248, Adjusted R-squared: 0.4984  F-statistic: 19.88 on 1 and 18 DF, p-value: 0.0003038  > summary(model3)$coefficient  Estimate Std. Error t value Pr(>\|t\|)  (Intercept) 16.7047222 7.512302 2.223649 0.0392126878  COD_initial -0.0976075 0.021894 -4.458185 0.0003037785 |
